# Evaluation of a shortened cardiac MRI protocol for left ventricular examinations: diagnostic performance of T1-mapping and myocardial function analysis

**DOI:** 10.1186/s12880-019-0358-9

**Published:** 2019-07-24

**Authors:** Jonathan Nadjiri, Anna-Lena Zaschka, Alexandra S. Straeter, Andreas Sauter, Maximilian Englmaier, Florian Weis, Karl-Ludwig Laugwitz, Ernst J. Rummeny, Daniela Pfeiffer, Michael Rasper

**Affiliations:** 1 Department of Diagnostic and Interventional Radiology, Klinikum rechts der Isar, School of Medicine, Technical University of Munich, Ismaninger Str. 22, 81675 Munich, Germany; 2 Department of Cardiology, Klinikum rechts der Isar, School of Medicine, Technical University of Munich, Ismaninger Str. 22, 81675 Munich, Germany

**Keywords:** Cardiac MRI, Economic, Shortened protocol, T1mapping, CMR

## Abstract

**Background:**

In this study we sought to retrospectively evaluate whether a very brief cardiac magnetic resonance imaging (CMR) protocol sufficiently distinguishes patients with relevant myocardial changes with need for further examination from healthy subjects.

**Methods:**

Patients with clinical indication for CMR (*n* = 160) were included in the study. Patients were categorized into two groups depending on presence of left ventricular (LV) dysfunction. ROC-analysis was done for results of T1-, T2- mapping and extracellular volume evaluation in patients without LV dysfunction. Binary endpoint was correctly depicted pathology of the conventional qualitative CMR techniques and report.

**Results:**

In the patient cohort without LV dysfunction (49%), AUC for T1 mapping was 82% (*p* < 0.001), 60% for T2 mapping (*p* = 0.1) and 79% for ECV (*p* < 0.001). T1 mapping was significantly superior to T2 mapping to rule out left ventricular pathology (*p* = 0.012). Sensitivity for the combined use of T1 mapping and sBTFE cine imaging was 98%; the negative predictive value was 90%. In 49 patients (30%) full protocol CMR did not provide any additional information; T1 mapping correctly detected 57% of the subjects from this group who would not benefit from additional CMR.

**Conclusion:**

A shortened CMR protocol comprising T1 mapping and LV-function analysis seems suitable to rule out myocardial alterations. Every third patient of the study population did not benefit from full contrast enhanced CMR. The shortened protocol correctly identified every fifth patient who would not benefit but no relevant pathologic findings with the obligation for treatment were missed.

## Background

Cardiac MRI (CMR) is a very established technique in clinical practice. However, the examination remains time consuming and requires expertise of both the technician and the reader since conventional techniques such as T2 weighted imaging and Late-Gadolinium-Enhancement mainly allow for qualitative or semi-qualitative analysis [1–3]. Depending on the given routine in a CMR laboratory, an examination to rule out structural heart disease or myocarditis can take 30 min up to an hour or more [4]. This limits the number of CMR examinations that can be offered to referring physicians. As a consequence, patients might be scheduled for CMR much later than the onset of symptoms delaying correct diagnosis and possibly reducing sensitivity of the examination due to chronification [4, 5]. Therefore, short examination times in CMR are mandatory to offer this highly potent diagnostic test to a broad population of patients with clinical indication. This is especially relevant for clinical routine use with an inhomogeneous patient population with many subjects having a low pre-test probability for myocardial disease.

Quantitative approaches like mapping sequences are now established in daily routine CMR [1, 3, 6–8]. T1 and T2 mapping allow for a per voxel calculation of the absolute myocardial relaxation time eliminating the need of comparison with assumed healthy tissue. Both techniques show excellent diagnostic performance regarding specific myocardial diseases such as infarction or myocarditis as well as storage diseases. Additionally, these sequences also bear prognostic value [9–11].

Native T1 mapping is able to detect edema, hemorrhage, siderosis, lipid and protein disposition as well as fibrosis [6, 12–14]. This overlap of different myocardial changes being detected by T1 mapping indicates a potential for utilization as a search sequence [7, 15, 16].

The purpose of the present investigation was to retrospectively evaluate whether a very brief CMR protocol sufficiently distinguishes patients with relevant myocardial changes with need for further examination from healthy subjects.

## Methods

### Study population

All consecutive patients receiving clinically indicated CMR for left ventricular evaluation between October 2015 and October 2017 were retrospectively evaluated. The study has been approved by the local ethics committee of the Medical Faculty of the Technische Universität München. Patients with missing T1 mapping were excluded as well as patients with evaluation of cardiac masses. All patients in this study received full contrast CMR.

### Imaging

Cardiac imaging was performed using a 3.0 T Philips Ingenia clinical dual-source RF transmission MR system (Philips Healthcare, Best, the Netherlands) as described before [17]. T1 mapping was performed as described before [17]. As a reference for mapping techniques, scans of healthy volunteers are highly recommended [7, 15, 16]. Mean T1 was 1175 ms measured in 13 healthy volunteers with the same protocol. T2 values in healthy volunteers were not available.

For the T2 mapping Gradient Spin Echo (GraSE) sequence, a train of spin-echoes is generated by several 180° radiofrequency pulses and each individual spin echo is acquired with an EPI readout. A six-echo variant was used. A dual inversion recovery black blood module was applied to null the signal of the blood. Electrocardiography-gated breath-hold GraSE sequences were acquired as follows: TR = 1 RR interval, TE 10–100 ms (9 echoes), flip angle 90°. Acquired voxel size was 2.0 × 2.0 × 8.0 mm3, matrix was 176 × 175 mm^2^. Besides T1 Modified Look Locker Inversion Recovery (MOLLI) and T2 GraSE imaging, all patients received a clinical cardiac MR protocol adjusted to the respective clinical issue. Cine sBTFE imaging, T2 TSE dark-blood imaging and Early-Gadolinium-Enhancement (EGE) (5 min after Gd application) and Late-Gadolinium-Enhancement (LGE) imaging (3D IR GRE or 2D PSIR) were performed in all subjects according to the institutional standard protocol. Dual source radiofrequency transmission was used for cine imaging; FOV was 320x410mm, voxel size was 1.9 × 1.19x8mm, TR was 2.6 ms and TE 1.2 ms. Flip angle was 45° and sense factor was 2. All images were obtained using retrospective gating. For all sequences the inline function arrhythmia rejection was used.

### Image analysis

LV-function analysis was done with sBTFE cine imaging in short axis view using the Philips Intellispace software (Ver. 8). LV-parameters were documented in the standard clinical CMR report. After review, this data was accepted as a study parameter. Myocardial evaluation was done using the AHA/ACC 17-segment model excluding segment 17 to avoid partial volume effects. Assessment of T2-weighted images, T1- and T2- relaxation time as well as extracellular volume (ECV) was based on corresponding short axes and measured for each of the abovementioned 16 segments. For that purpose, regions of interest (ROI) were semi-automatically delineated using the Philips Intellispace software (Ver. 8). For reference in T2w imaging an additional ROI within skeletal muscle (ROI > 30 mm^2^) was delineated. T2w images were semi-quantitatively assessed by calculation of ratio of signal intensity of myocardium and skeletal muscle as described before [18]. At our institution the local threshold for T2w ratio was defined with 2.5. Imaging for EGE, LGE and wall movement disorders were evaluated visually and dichotomously in sBTFE cine imaging. For calculation of ECV a synthetic haematocrit was estimated as proposed by Treibel et al. [19]; however, an adjustment using curve fitting was necessary to compensate for 3 T compared to 1.5:

$$ Estimated\kern0.3em haematocrit=\left(\frac{973}{Native\kern0.2em T1\kern0.3em relaxation\kern0.3em time\kern0.3em of\kern0.3em blood}\right)-0,1232. $$

ECV was calculated as described before [8, 20]. For analysis in every patient the segment with highest T1- and T2 relaxation times as well as ECV und T2 ratios were defined; all highest segments were then reviewed again. Segments with artefacts were carefully excluded and replaced by the segment with the second highest value without artefact. These variables were defined as T1max, T2max and ECVmax, respectively. Selection of the segments with highest values of T1, T2 and ECV allows to compare focal myocardial alterations with global ones. All clinical reports were reviewed by a Level III CMR reader (certified by the European Association of Cardiovascular Imaging).

For the study analysis CMR data was evaluated and ROIs were delineated by a CMR reader in training; this data was later again reviewed by a Level III CMR reader. As binary endpoint we defined no myocardial finding in the CMR report, normal signal characteristics in all standard sequences (T2w DB, EGE and LGE) and - depending on subgroup analysis - also LV-parameters. Pericardial effusion or thickening without myocardial involvement were not regarded as myocardial pathology and therefore categorized as no finding for this analysis. In case of equivocal findings definite decision was made by the Level III CMR reader.

### Statistical analysis

Continuous variables were expressed as mean ± standard deviations (SD). The tested data has visually been evaluated for normal distribution. Two-sided t test was utilized for exploratory testing for gender differences of native and contrast enhanced T1 relaxation. For assessment of correlation Pearson’s coefficient has been determined; values of 0.3–0.5 are regarded as low, 0.5–0.7 as moderate, 0.7–0.9 as high and values of 0.9–1 as very high correlation as suggested [21]. Receiver operator characteristic (ROC) analysis was performed using the method of DeLong [22]. As endpoint for ROC-analysis we defined no myocardial finding in the CMR report within the entire study population. In subgroup analyses in patients with no LV-dysfunction the endpoint was any myocardial pathologic finding in the CMR report excluding LV-parameters. And in subgroup analyses in patients with LV-dysfunction the endpoint was any further myocardial pathology besides LV-dysfunction. Optimal cut-off values were calculated by maximization of the sum of sensitivity and specificity. As global level of significance a *p* value of 0.05 was accepted. The statistical package R version 3.0.3 [23] was used for statistical analysis.

## Results

### Study population

**Fig. 1:**
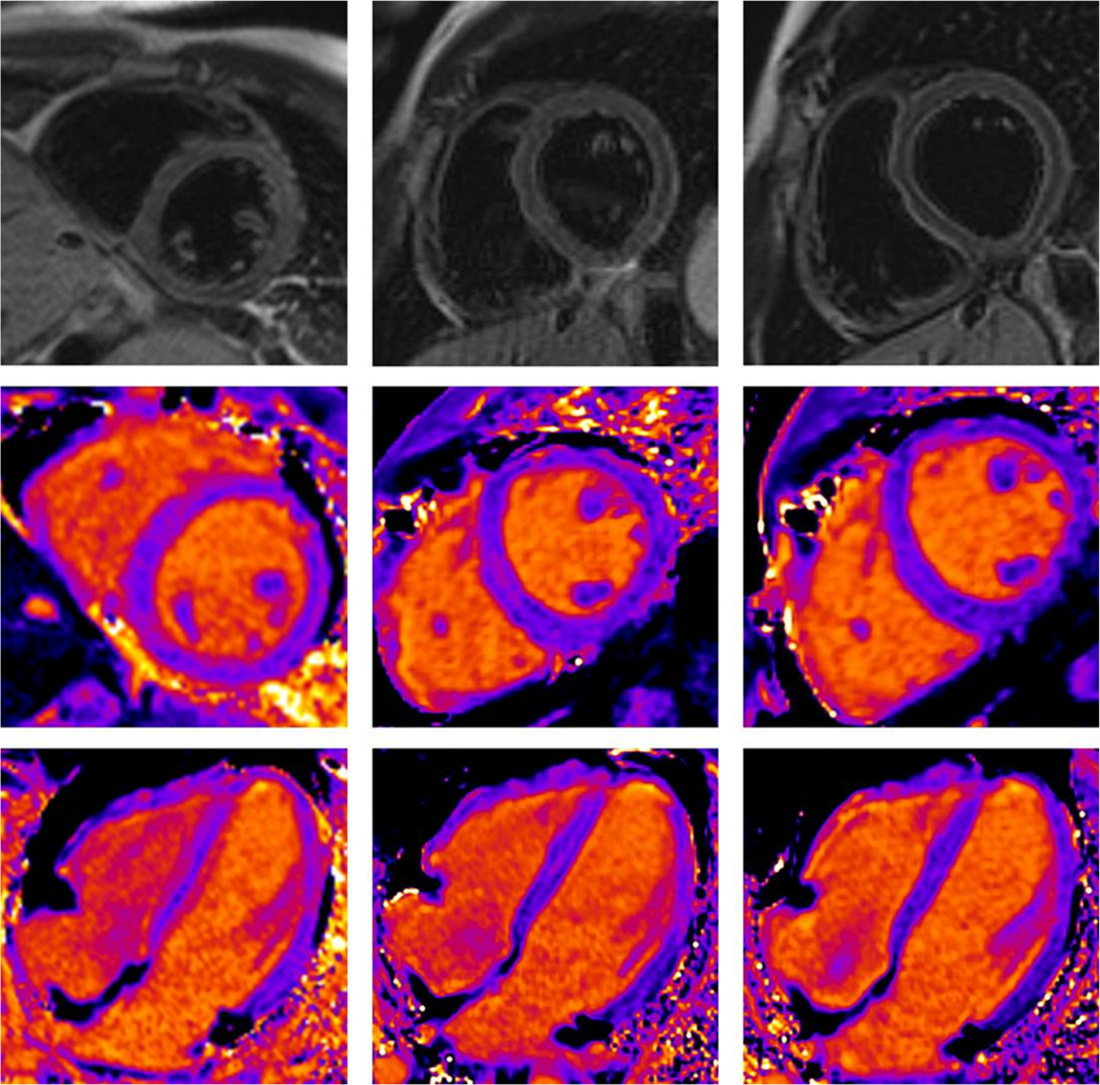
Example of an 18-year-old male patient with severe myocarditis. Native sequences: T2w dark blood (top row) and native T1 mapping in short axis (middle row) and 4 chamber view (lower row) at acute state,8 and 16 weeks follow up exam (columns from left to right). Highest T1 relaxation time in this patient was 4 standard deviations above values of volunteers and well above our threshold in acute state and within normal range at follow-up. It is of note that T2w imaging appears to be normal due to inflammatory involvement of the whole heart. Even the ratio of myocardium and skeleton muscle signal intensity was normal as a result of accompanying myositis

**Fig. 2:**
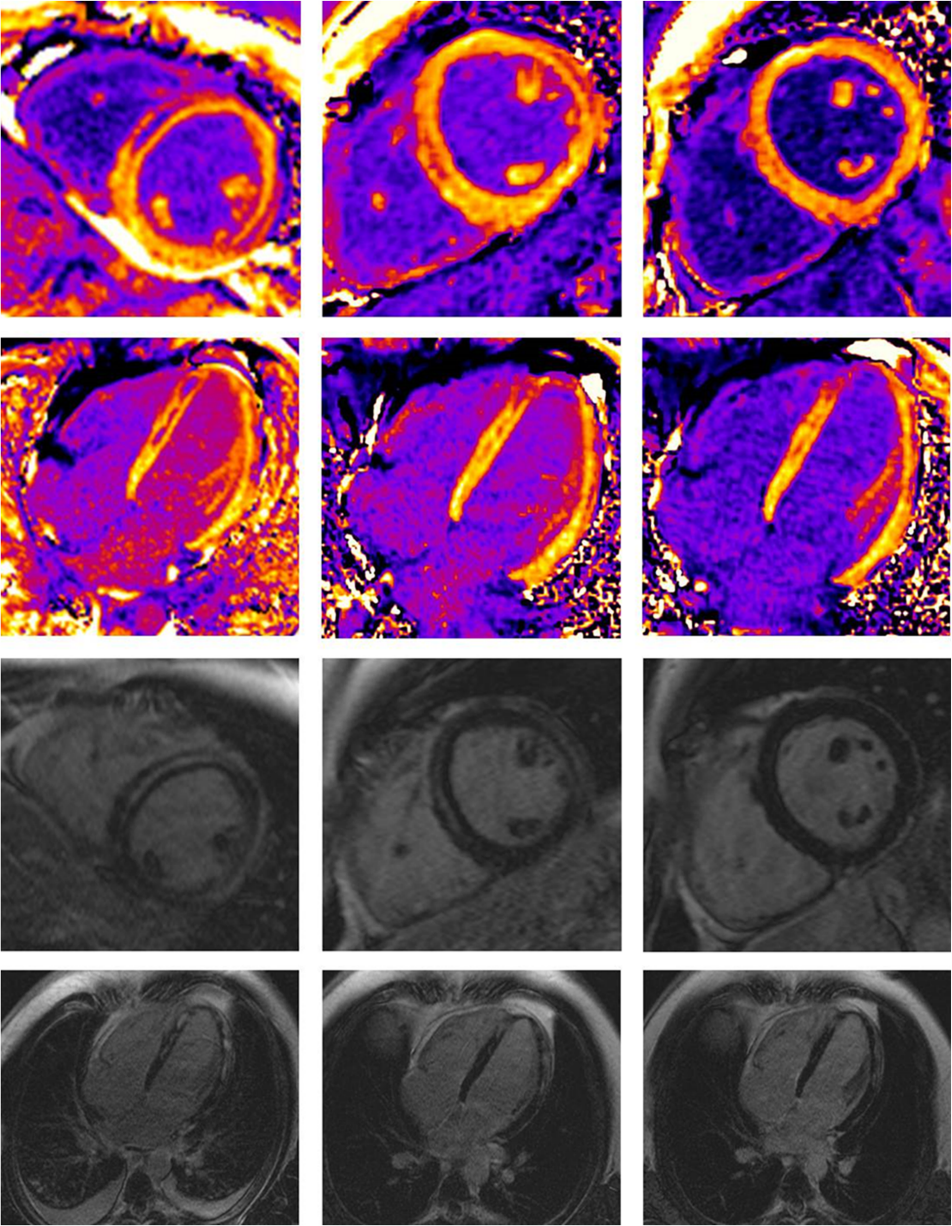
Example of an 18-year-old male patient with severe myocarditis. Contrast enhanced sequences: CE T1 mapping in short axis and 4 chamber view (first two rows) and Late-Gadolinium-Enhancement (last two rows) at acute state and 8 and 16 weeks follow up exam (columns from left to right). Maximum ECV was 51% in acute state in the anterior septal wall and 25% at 16 week follow up

In total 160 patients were included in the study. 66% of the patients were male. Mean age at examination was 45 ± 16 years. In Table 1 a breakdown of CMR indications is provided. In Figs. 1 and 2 image examples of a severe case of myocarditis with follow-up is provided.

**Table 1:** Lists general patients’ information with a break down of the Cardiac MRI (CMR) indication written on requesting form

Patients information (*n* = 160)	
General information	
Age	45 ± 16 years
Male gender	106 (66%)
Synthetic haematocrit	0.41 ± 0.028
Indication for CMR	
Acute myocarditis	81 (51%)
Chronic myocarditis	13 (8%)
Structural heart disease	9 (6%)
Unspecific clinical indications (e.g. check-up)	21 (13%)
Systemic disease	19 (12%)
Ischemic heart disease	17 (11%)

### CMR findings

In 69% (*n* = 111) of patients there was a positive finding in CMR. 51% (*n* = 82) had either focal or global left ventricular (LV) dysfunction. In 34% (*n* = 55) increased myocardial T2w signal was found. In 43 patients (*n* = 27%) EGE was present. In 40% (*n* = 65) of patients positive LGE was found. In Table 2 a breakdown of CMR diagnosis as documented in the final CMR report is provided. Table 2 shows further findings from CMR in more detail. All diagnoses were made with final consensus and agreement of the department of Cardiology at daily conferences.

**Table 2:** Lists general patients’ CMR information together with a breakdown of the diagnoses reported by cardiac MRI (CMR)

CMR data (*n* = 160)	
LV parameters	
Endsystolic volume	74 ± 53 ml
Enddiastolic volume	158 ± 60 ml
Ejection fraction	55 ± 12%
Regional wall motion abnormality	36 (23%)
Myocardial characterisation	
Mean T1 relaxation time^a^	1266 ± 89 ms
Maximum T1 relaxation time^b^	1343 ± 98 ms
Number of segments with increased T1 relaxation time(> 1300 ms[first quartile of the study population])	2.5 ± 4.1
Mean T2 relaxation time^a^	49.7 ± 5.4 ms
Maximum T2 relaxation time ^b^	58.1 ± 9.7 ms
Number of segments with increased T2 relaxation time (> 55 ms)	2.3 ± 3.9
Mean ECV^a^	30.6 ± 4.6%
Maximum ECV^b^	34.3 ± 5.5 ms
Number of segments with increased ECV relaxation time (> 34%)	4.3 ± 5.4
Number of segments with increased signal intensity in T2w DB	1.8 ± 2.7
Positive EGE	43 (27%)
Positive LGE	65 (41%)
Diagnosis from CMR report	
Acute myocarditis	19 (12%)
Chronic myocarditis	21 (13%)
Structural heart disease	18 (11%)
Unclear or unspecific diagnosis	26 (16%)
Systemic disease	15 (9%)
Ischemic heart disease	12 (8%)
No finding in CMR report or minor unspecific findings (e.g. slightly increased amount of pericardial fluid)	49 (31%)

^a^ average of segment 1–16, ^b^ segment with highest value

### Results of ROC analyses - comparison of the sequences

#### ROC analysis in the entire study population (*n* = 160)

Area under the curve (AUC) for native T1 mapping evaluating the segment with highest T1 relaxation time (T1max) was 78%, for ECV (ECVmax) 73%; *p* < 0.001, 95% CI [71–85% and 65–81%]. Optimal cut-off was 1335 ms for native T1 mapping with a sensitivity of 67% and a specificity of 67%. Best cut-off for ECV was 34% with a sensitivity of 67% and a specificity of 67%. Sensitivity for the combined use of T1 mapping and sBTFE cine imaging was 97%. Additional T2 mapping was available for 110 patients: ROC was 69%; *p* < 0.001, 95% CI [59–78%]. Sensitivity was 72% and specificity was 60% with optimal cut-off of 55 ms.

#### ROC analysis in patients with (*n* = 82) and without (*n* = 78) LV dysfunction

In patients with LV dysfunction AUC was 74% for T1max and 73% for ECV; *p* < 0.001 for both, 95% CI [61–87% and 59–88%]. Optimal cut-off for T1max was 1346 ms with a sensitivity of 60% and specificity of 63%. For ECVmax in patients with LV dysfunction optimal cut-off was 34% with a sensitivity and specificity of 70 and 69% respectively.

In patients without LV dysfunction AUC for T1max was 82 and 75% for ECVmax; *p* < 0.001 for both. For difference of the curves p was 0.25. Optimal cut-off for T1max was 1336 ms and 34% for ECV. Sensitivity and specificity for T1max was 74 and 74%. For ECVmax sensitivity and specificity was 67 and 68%.

#### Comparison in patients with no LV dysfunction and additional T2 mapping (*n* = 56)

AUC was 82% for native T1 mapping evaluating the segment with highest relaxation times; *p* < 0.001, 95% CI [71–94%]. Optimal cut-off was 1338 ms with a sensitivity of 77% and a specificity of 74%.

For calculated ECV, AUC was 79% for the segment with highest value; *p* < 0.001, 95% CI [66–92%]. Optimal cut-off was 34% with a sensitivity of 69% and a specificity of 70%.

AUC was 60% for T2 mapping (T2max) for the segment with maximum relaxation time, *p* = 0.1, 95% CI [45–76%]. Optimal cut-off was 55 ms with a sensitivity of 54% and a specificity of 60%.

**Fig. 3:**
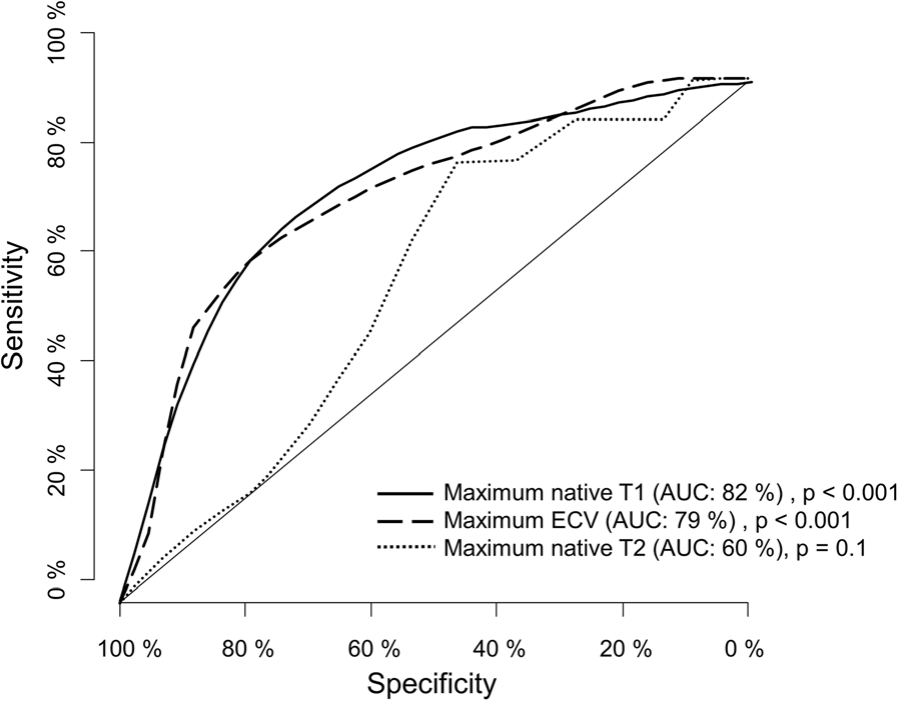
shows a ROC analysis of the three quantitative techniques (T1 mapping [solid line], T2 mapping [dotted line] and extracellular volume [dashed line]) in patients with no left ventricular dysfunction where additional T2 mapping was available (*n* = 56)

Difference of the AUC was 22% in favour of T1max over T2max; *p* = 0.012. For T1max the difference of the AUC was 3% in comparison to ECVmax; *p* = 0.7. Difference of AUC between T2max and ECVmax was 19% in favour of ECVmax; *p* = 0.013. ROCs for the three quantitative sequences are provided in Fig. 3.

### Analysis of individual benefit from full contrast CMR

**Fig. 4:**
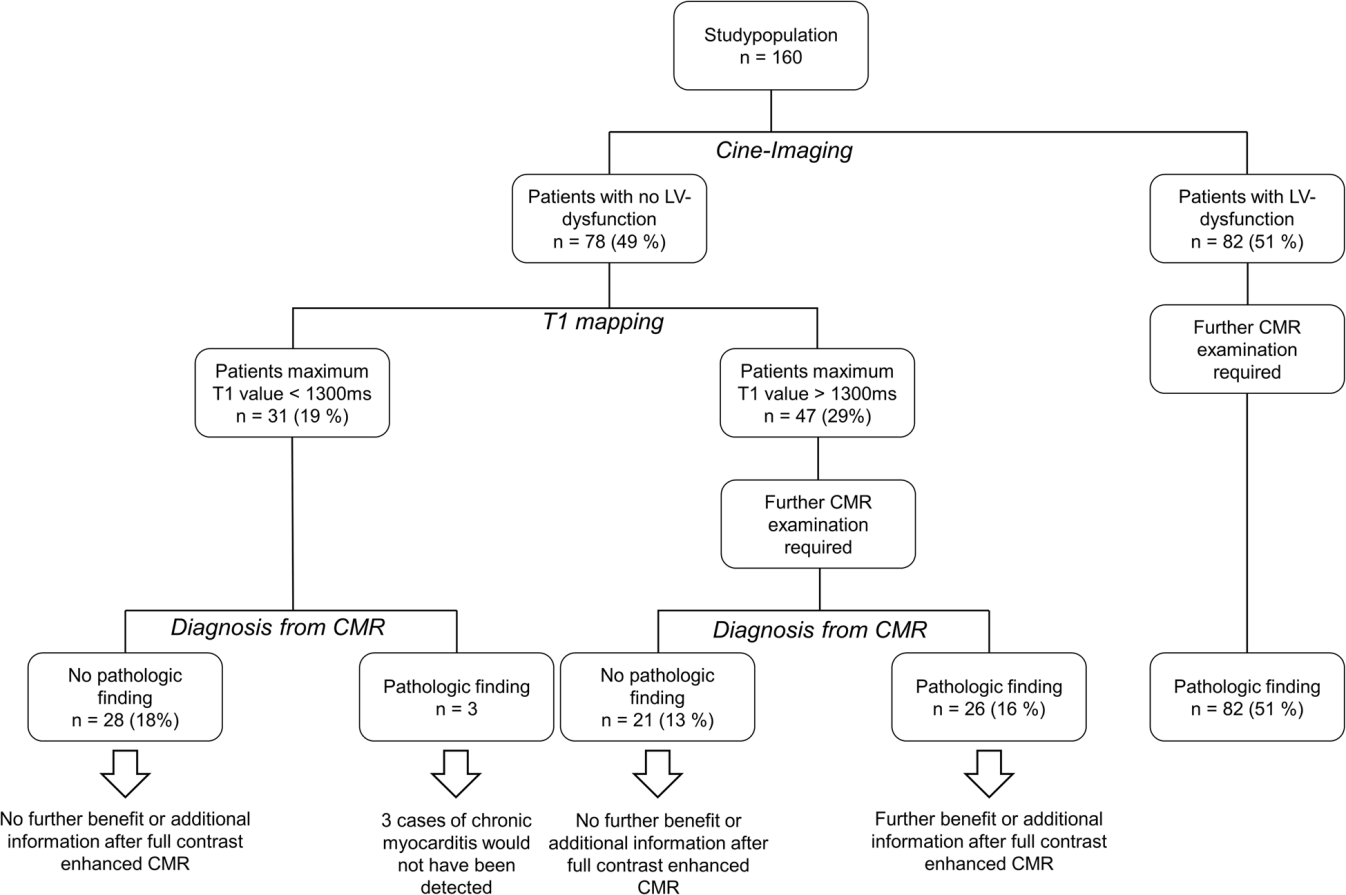
shows the flow chart of diagnostic outcomes in our study population based in presence of LV dysfunction and T1 relaxation times. All patients received full contrast CMR. It further illustrates the groups that benefit form full contrast enhanced CMR and also those who do not. The discrimination of this groups is also shown for a threshold value of T1 mapping (1300 ms)

In our study 51% (*n* = 82) patients had LV dysfunction and therefore requiring full contrast enhanced CMR. 49% (*n* = 78) of the patients showed no LV dysfunction. In this cohort 40% (*n* = 31) had a T1max of less than 1300 ms (first quartile of the study population) and thus below the optimal cut-off. In this group 90% (*n* = 28) had no pathological finding in their final report and therefore did not benefit from the full contrast CMR protocol. In the group of patients with a T1max below 1300 ms, 10% (*n* = 3) had a pathologic finding; all three cases were consistent with chronic myocarditis. From the cohort with T1max higher than 1300 ms 45% (*n* = 21) had no findings in the CMR report, thus the full CMR exam did not provide any additional information. However, from the same cohort with T1max higher than 1300 ms 55% had a pathologic finding and therefore benefitted from full contrast CMR. In our study population 70% of the patients (*n* = 111) had a finding in their final CMR report and 30% (*n* = 49) did not. Of these 49 patients 57% (*n* = 28) could be identified as patients who would not benefit from full contrast CMR by T1 mapping and cine imaging alone. In patients without LV dysfunction the negative predictive value of native T1 mapping was 90%. A detailed illustration of the examination outcome is provided in Fig. 4.

## Discussion

The purpose of the present investigation was to evaluate a shortened CMR protocol in clinical routine practice. Therefore, a very heterogenous patient collective was investigated. The main findings of this study are i) T1 mapping is superior to T2 mapping in detection of unspecific myocardial pathology in a heterogenous study population of patients without LV-dysfunction and ii) a shortened protocol comprising only T1 mapping and LV-function analysis discriminates patients who will benefit from a full contrast enhanced CMR protocol from those who do not with sufficient confidence.

### Need for shorter examinations

In clinical practice CMR, amongst a few other applications, is considered to be the most time consuming and therefore economically challenging examination. Due to lengthy protocols, emergency examinations cannot easily be integrated into a busy schedule even if there are drop outs. Further, the examination is technically challenging and labor intensive. In an optimal setting a CMR examination occupies one trained physician and up to two technicians [4, 24]. This further stresses an effective and economic use of MRI scanners. Long examination times also hamper patient comfort. As a consequence, important CMR examinations can be delayed in the clinical routine while CMR reports are often urgently needed for clinical decision making [5]. Therefore, shorter CMR protocols are desirable allowing more patients to benefit from the high diagnostic value that makes CMR the diagnostic gold standard in detection of several cardiovascular diseases. Yet, data on rigorously shortened CMR protocols is very rare.

### Evaluation of the study results

The suggested protocol in this study comprises a quantitative approach for myocardial assessment and a combination of quantitative and qualitative evaluation of the left ventricle. In our study population T1 mapping was superior to T2 mapping and was therefore further investigated in the shortened CMR protocol. Prolongation of T1 relaxation time can be found in several entities of myocardial pathology such as edema, fibrosis, protein deposition and others [7]. In contrast, T2 relaxation times are mainly increased in presence of edema and slightly in case of fibrosis. Additionally, a broad range of myocardial T2 values can be found in healthy volunteers complicating to set a strong threshold value [25, 26]. It is to assume that those are the two main reasons why T1 mapping was superior to T2 mapping in detection of patients with a pathological finding in the final CMR report in our heterogenous study population. Further, we also found good diagnostic performance of ECV evaluation. However, determination of ECV requires T1 mapping measurement before i.v. gadolinium application and approximately 10 min after. For that reason, this technique does not necessarily lead to a shortened examination time. Yet, native T1 mapping was not inferior to ECV measurement in our population; this is of note since the latter is a contrast enhanced technique with partially inherent information about EGE/LGE [27]. Additionally, non-enhanced protocols are desirable due to the growing knowledge on deposition of MR contrast agents in the human body [28, 29] and increasing concerns against Gd application on the patients’ side.

One approach to shorten CMR examination time is to predict at an early state which patient will benefit from the full contrast enhanced examination and which will not. In our collective every third patient did not benefit from a full contrast enhanced CMR. 57% of those subjects were correctly identified by native T1 mapping and cine imaging alone. Regarding the entire study population, the shortened protocol correctly ruled out pathology in every fifth patient.

Yet, three cases of pathologic findings were overlooked using the shortened sequence protocol. Those three cases were consistent with chronic myocarditis. It is of note that this diagnosis does not represent acute myocardial damage with inherent treatment indication. Therefore, clinical relevance is questionable if there is no other pathologic myocardial alteration present like LV dysfunction or others. Additional T2 mapping would not have detected these 3 cases of chronic myocarditis. In summary, no case of acute myocardial damage with inherent obligation to treat has been missed.

### Future applications of the shortened protocol – can it be used as a screening method?

Even though the proposed shortened CMR protocol yielded satisfying results, a standardized use in clinical routine is somewhat challenging. Application as a screening protocol with image analysis after the scan would neither be cost nor time effective since approximately 70% of the patients would have to be rescanned with the need for full contrast CMR. Although a partial scan has already been acquired and those sequences could be omitted in the full contrast scan, positioning of the patient in the scanner and geometrical planning of the sequences is a very time-consuming part of the examination. As a result, net scanner occupancy would not be reduced.

Another approach would be to immediately evaluate the image data including determination of myocardial T1 values as well as LV-parameters while the patient is still inside the scanner. If both T1 relaxation time and LV functional parameters are within normal range, further sequences and contrast application can be omitted with fair confidence. According to our mixed cohort, this will assumably be the case in approximately every fifth patient. The saved scanning time might be used to increase the number of CMR examinations offered to referring physicians.

The disadvantage is a reduced predictability of the scan length. Further, this also requires a physician permanently present at the scanner to be effective.

Assuming that a full protocol takes 60 min and the proposed shortened protocol 30 min, the second scenario would allow to stop after the shortened protocol in 31 cases from our study population. This would safe 15.5 h (10%) of scanner time at the cost of 2% incorrect diagnoses only comprising chronic myocarditis of which clinical relevance and evidence-based treatment consequence, respectively, remain unclear.

Additionally, the presented findings might be of importance when shortened protocols are a necessity instead of a choice, for example when the patients’ clinical condition does not allow for a time consuming full CMR protocol or if image quality of contrast enhanced sequences is hampered due to technical issues. Our study results indicate that in these cases the radiologist can rely on cine and mapping imaging for making a diagnosis.

Besides the economic relevance of the findings of this study, the diagnostic potential of the proposed shortened protocol has to be further evaluated prospectively in a larger multicentric study population.

It is of note that in this study sBTFE cine imaging was used for LV evaluation. Real-time imaging could further strongly shorten examination time. Recent studies have shown that measured values showed high accuracy and reliability [30]. Therefore, a protocol comprising only T1 mapping and real-time cine imaging seems very promising since examination time can probably be reduced to less than 15 min.

### Limitations

This is a retrospective single centre study. The study results are limited due to the relatively small size of the study population. Even though, the purpose of the study was to evaluate the shortened protocol for the left ventricle in daily practice conditions and therefore requiring a heterogenous cohort the composition of the study population might be dependent of statistic variations as might the subsequent results. Further, no validation against solid markers was available (e.g. laboratory values such as troponin or tissue from myocardial biopsy). In the study population right ventricular pathology was excluded. The shortened protocol would assess RV-wall movement disorders but the spatial resolution of the proposed T1 mapping sequence is probably too low to allow full and correct diagnosis on the RV limiting the method to LV evaluation.

## Conclusions

A shortened CMR protocol comprising only T1 mapping and LV function analysis seems to sufficiently rule out myocardial alterations in our study. Sensitivity was 98%; only three cases of chronic myocarditis with normal LV-function were overlooked, yet therapeutic consequences of this entity remain uncertain. The proposed protocol might enhance cost-effectiveness by shorter examination time and by omitting contrast application without missing relevant findings for certain clinical questions, especially when pre-test probability is low. Shorter examination times optimize patients’ comfort.

## Data Availability

The datasets used and/or analysed during the current study are available from the corresponding author on reasonable request.

## Abbreviations

AUC: Area Under the Curve
CMR: Cardiac Magnetic Resonance imaging
DB: Dark Blood
ECV: Extra Cellular Volume
ECVmax: Highest Extra Cellular Volume
EGE: Early-Gadolinium-Enhancement
EPI: Echo-planar imaging
GraSE: Gradient Spin Echo
GRE: Gradient Echo
IR: Inversion Recovery
LGE: Late-Gadolinium-Enhancement (LGE)
LV: Left Ventricle
MOLLI: Modified Look Locker Inversion Recovery
ms: milliseconds
PSIR: Phase Sensitive Inversion Recovery
ROC: Receiver Operator Characteristic
ROI: Regions Of Interest
sBTFE: Sense Balanced Turbo Field Echo
SD: Standard Deviation
T1max: Highest T1 relaxation time in ms
T2max: Highest T2 relaxation time in ms
TE: Echo Time
TR: Repetition Time
TSE: Turbo Spin Echo
